# Editorial: Immunotherapeutic and Immunoprophylactic Strategies for Infectious Diseases

**DOI:** 10.3389/fimmu.2020.01670

**Published:** 2020-07-28

**Authors:** Giuseppe A. Sautto, Roberta A. Diotti

**Affiliations:** ^1^Center for Vaccines and Immunology, University of Georgia, Athens, GA, United States; ^2^Microbiology and Virology Unit, “Vita-Salute San Raffaele” University, Milan, Italy

**Keywords:** infectious diseases, vaccines, antiviral drugs, monoclonal antibodies, chimeric antigen receptor (CAR)

The necessity of a rapid development of effective therapeutic and prophylactic strategies for infectious diseases has recently gained further attention and importance due to the very recent SARS-CoV-2 pandemic (1). In fact, these countermeasures are of pivotal importance not only and principally for reducing the associated diseases, deaths, and overload of hospitalized patients during the pandemic outbreak, but also in limiting the tremendous impact on the economy and the society.

It is also important to highlight the fact that while COVID-19 is nowadays a central health issue attracting the attention of a significant portion of the scientific community as well as enormous financial aids, other infectious diseases still represent a global burden for the world population. As an example, influenza cases are still characterized by high morbidity (30–50 million cases yearly) and mortality rates. Approximately 3 to 5 million of these cases are characterized by a severe illness and about 290,000 to 650,000 respiratory deaths are reported annually, according to the World Health Organization (WHO). Additionally, the possibility of new influenza pandemic outbreaks aggravating these case numbers still represents a considerable risk.

In this regard, implementing current vaccination strategies, such as in the case of the current Yellow Fever vaccine as described in a paper of this article collection by Campi-Azevedo et al, and designing and developing next-generation vaccines, especially for high-risk populations, like in the case of influenza (Mathew and Angeletti), represent an important goal for the investigators working in this field not only to limit the related diseases but also to alleviate the associated economic burdens.

Importantly, novel immunotherapeutic strategies are also pivotal to reduce the severity and improve the current drug arsenal to combat influenza infections (De Vlieger et al.), which is currently limited to only two main approved antiviral drug categories: the neuraminidase and the M2 ion channel inhibitors.

In addition to influenza, other respiratory pathogens, such as bacteria and viruses, are responsible for a considerable number of infection cases, especially in more vulnerable subjects, such as children, the elderly, and immunocompromised individuals. Among these pathogens, pneumoviruses represent the leading cause of viral bronchiolitis and viral pneumonia in infants and children that can also result in a fatal outcome. It is thus important to improve our knowledge of the molecular mechanisms leading to a successful immune response, such as the studies aimed at dissecting the antibody response to the main pneumovirus surface fusion proteins (Huang et al.). Furthermore, a comprehensive understanding of the immunomodulatory properties exerted by bacterial pathogens, such as *Mycobacterium tuberculosis* (MTB) and *Bordetella* spp., could help in designing and developing more effective therapeutic and prophylactic strategies aimed at eliciting an effective immune response (Gestal et al.). In particular, these studies are also crucial for developing alternative immunotherapeutic strategies as well as for the design of effective vaccines not only for infectious diseases but also for other disorders, such as autoimmune diseases (Takaya et al.).

As far as bacterial respiratory infections, MTB is certainly one of the most diffused respiratory pathogens worldwide, representing one of the top 10 causes of death and the leading cause from a single infectious agent, according to WHO. Developing new therapeutic strategies to overcome the occurrence of multidrug-resistant strains as well as effective vaccines able to prevent and limit the progression of the lung pathology in infected patients, represents a top health priority worldwide, especially for developing countries. As an example, monoclonal antibodies (mAbs) represent a valid immunotherapeutic approach to target multidrug resistant pathogens (2). Importantly, in recent years, the mAb discovery field has encountered an outstanding renovation and innovative development, mainly thanks to the advancement and improvement of next-generation sequencing (NGS) approaches at the single-cell level. In this regard, the “omics” technologies, employing large genomic, transcriptomic, structural, and proteomic datasets and the interpretation of them under a systems biology paradigm (Martín-Galiano and McConnell), allow for the rational identification of rare mAbs along with the deconvolution of their functional profile (3). Importantly, besides the direct use of mAbs as therapeutics, or as recently proposed, as prophylactic molecules to prevent infection in a determined period time, such as during influenza seasons, they can also be utilized as a tool or engineered for the development of prophylactic or cell-mediated anti-infective strategies, respectively (4). In this regard, the success of chimeric antigen receptor (CAR) T cell therapy for the treatment of difficult to eradicate cancers has inspired researchers to develop CARs for the treatment of infectious diseases as a potential therapeutic option for patients who are unresponsive to standard treatments (Seif et al.).

Different prophylactic strategies are currently under development and targeting different antigens expressed at different stages of bacterial (e.g., MTB) (Kwon et al.) as well as parasitic pathogens (e.g., *Plasmodium* and *Trichinella* spp.) (Stachyra et al.). In this regard, a lot of currently under development prophylactic strategies are also designed not only for prevention purposes but have also been proposed as therapeutic vaccines aimed at boosting or eliciting a *de novo* immune response to eradicate or mitigate infections. In this context, vaccines for human papillomavirus (HPV) have been described to be possibly effective also in the treatment of HPV-related lesions and relapse (Garbuglia et al.).

Additionally, developing novel immunotherapeutic and immunoprophylactic approaches that can be possibly delivered at the site of inflammation (Qin et al.; Zhang et al.) or infection, such as through inhalation or oral administration, improving their bioavailability and efficacy characteristics, represent a valid and desirable strategy for respiratory infections, including MTB (Sécher et al.).

As shown in the review and research articles of this collection, as well as in the recent literature, thanks to the new technologies and rapid scientific advancements it is now possible to expedite the research of innovative prophylactic and therapeutic countermeasures, possibly reducing the time of their approval in the clinical practice. This is certainly true in an emergency setting, like in the course of a pandemic event, but it could be applicable in the near future, where personalized, more effective, specific and rapid interventions will be employed in the clinical routine.

## Author Contributions

All authors listed have made a substantial, direct and intellectual contribution to the work, and approved it for publication.

## Conflict of Interest

The authors declare that the research was conducted in the absence of any commercial or financial relationships that could be construed as a potential conflict of interest.
